# Editorial: Real-World evidence in onco-hematological patients

**DOI:** 10.3389/fonc.2022.1060802

**Published:** 2022-11-07

**Authors:** Claudia Vener, Matteo Franchi, Annalisa Trama

**Affiliations:** ^1^ Department of Oncology and Haemato-Oncology, University of Milan, Milan, Italy; ^2^ National Centre for Healthcare Research and Pharmacoepidemiology, University of Milano-Bicocca, Milan, Italy; ^3^ Laboratory of Healthcare Research & Pharmacoepidemiology, Department of Statistics and Quantitative Methods, University of Milano-Bicocca, Milan, Italy; ^4^ Department of Research, Evaluative Epidemiology Unit, Fondazione Istituto di Ricovero e Cura a Carattere Scientifico (IRCCS) Istituto Nazionale dei Tumori, Milan, Italy

**Keywords:** haematological malignancies, clinical practice, real-world evidence, representativeness - generalizations, confounding (epidemiology)

Clinical practice, particularly in onco-haematological settings, relies on *randomised controlled trials* (RCTs), which provide rigorous scientific evidence under controlled experimental conditions. RCT results cannot be simply generalised to everyday clinical practice because of low overall trial accrual (<5% of all newly diagnosed cancer patients) and under-representation of patient frailties, as older age, advanced disease, concurrent disorders, lower socio-economic status, racial and ethnic minority membership, gender (females are less represented), and pregnancy. Conversely, *real-world studies* generate evidence on the actual benefits achieved in real-life settings, an essential requirement for public health research designed to assess and improve the impact of daily life treatment.

Our Research Topic confirms the importance of real-world studies by providing data on the pattern and quality of care and access to adequate healthcare, required to inform healthcare organisation. Interestingly, Pajiep et al. used administrative data from healthcare administrative databases in France, between 2011 and 2014, to build a specific algorithm to identify new cases of chronic myeloid leukaemia, describing patterns of tyrosine kinase inhibitor use and healthcare consumption. Daneels et al. adopted an innovative approach to describe patterns of care for diffuse large B-cell lymphomas based on Belgian health insurance data, underlining the importance of including old patients.

From a clinical viewpoint, *real-world studies* have permitted the study of therapy-related late effects. Trama et al. highlighted that survivors of adolescent and young adult haematological cancers face persistent long-lasting risk for many diseases, warranting careful consideration in cancer surveillance. Interestingly, Xiao et al. revealed substantial racial and ethnic differences associated with second malignant neoplasm subtype, risk, and mortality among Hodgkin lymphomas to be closely evaluated in cancer surveillance, and stressed the importance of including minorities in future studies. Originally, Efficace et al. presented preliminary results, provided by treating haematologists, on the clinical utility of integrating electronic patient-reported outcomes into daily practice.

Moreover, *real-world studies* enable the study of advanced disease (Liu et al.,  Lecat et al.), comorbidities (Jia et al.), rare haematological cancers due to the huge amount of collected cases (Zhu et al., Liu et al.), prognoses (Vener et al.,  Daneels et al.,  Corley et al.,  Lecat et al.), and permit model prediction (Morabito et al., Jia et al.). *Real-world studies* also allow us to confirm in real-life what has been observed in other settings (Li et al.).

Despite the huge potential of real-world data in monitoring and evaluating healthcare patterns, including diagnosis, therapy, assistance, and rehabilitation in daily clinical practice, some methodological issues remain the subject of debate and are discussed in this Research Topic.

First, real-world studies are often based on small monocentric studies, limiting the representativeness of the patients included in the study cohort and the generalisability of the results. Second, as in all observational studies, when comparing individuals subject to two or more exposure levels, cohort patients are not randomised, making real-world studies susceptible to confounding. Indeed, exposed and unexposed patients differ for several measured or unmeasured characteristics outside the exposure of interest, which can bias the observed measures of association between exposure and outcome. This issue is particularly critical in studies using secondary data, collected for purposes other than clinical practice, which consequently do not include detailed clinical and behavioural information. Third, the criteria for defining the exposure or outcome of interest are not always objective. For example, the definition of progression-free survival is based on algorithms which have not been validated, generating unknown errors (i.e. false negative and false positive outcomes) associated with their use. Moreover, the frequent lack of detailed data on both administered therapies and clinical information may lead to misclassification of exposures and/or outcomes of interest.

Authors do not always take appropriate account of the aforementioned issues. These should instead be presented in the “Methods” section, where the criteria for defining exposures, outcomes, and covariates of interest are clearly described, and in the “Discussion” section, highlighting the study limitations, any potential associated bias, and the direction of the bias (for example, underestimation or overestimation of the association of interest). Articles should also adhere to RECORD reporting guidelines (1), used to describe studies adopting routinely collected, observational data. Furthermore, before being carried out, we believe that observational studies, particularly ones based on secondary data, should be examined and approved by an Ethics Committee, to guarantee that the study will be conducted according to best observational research practice.

Furthermore, issues of data accessibility and delays in data availability, intrinsic to retrospective data, do not always allow prompt evaluation of the clinical impact of new interventions.

Hence, what steps should, in our view, be taken to advance the development of real-world evidence?

1) Exploit real-world data by integrating the many heterogeneous datasets available (e.g. administrative datasets with population-based cancer registries and data collected in electronic medical records) to increase information potential and data representativeness. The knowledge that can be acquired from combined data could not be derived from any single source. However, heterogeneous environments also contain several biases that need to be addressed with new analytical tools (2).

2) Leverage novel artificial intelligence (AI) approaches to allow information extraction from unstructured data (e.g. electronic medical notes).

3) Ensure data exchange among multiple data sources and repositories by exploiting emerging common data models (CDM) (https://www.ohdsi.org/data-standardization/the-common-data-model/; https://build.fhir.org/ig/HL7/cdmh/). CDM enable information (e.g. encounters, patients, diagnoses, drugs, measurements, and procedures) to be captured uniformly across different data sources.

4) Facilitate data re-use and sharing by implementing data exchange and altruism concepts (i.e. voluntary data sharing for the benefit of citizens) aligned with both existing and emerging EU policies. Data sharing is still limited by a number of stumbling blocks (e.g. low trust in data sharing, issues with public sector data re-use, data collection for the common good, and technical obstacles).

5) Support communities of practice, internationally and nationally, to improve the use of real-world data (e.g. sharing of best practices, innovative tools for data exchange and harmonisation, AI-based analytics, etc.) through regular interaction.

6) Boost trust in real-world data by increasing the number and quality of studies and publications on real-world data; organising conferences focused on real-world data, and developing dedicated educational and training opportunities.

## Author contributions

CV, MF, AT equally contributed to conception and design of the editorial. All authors contributed to manuscript revision, read, and approved the submitted version.

## Conflict of interest

The authors declare that the research was conducted in the absence of any commercial or financial relationships that could be construed as a potential conflict of interest.

## Publisher’s note

All claims expressed in this article are solely those of the authors and do not necessarily represent those of their affiliated organizations, or those of the publisher, the editors and the reviewers. Any product that may be evaluated in this article, or claim that may be made by its manufacturer, is not guaranteed or endorsed by the publisher.
